# Supplementary material for: Challenges and Choices in Breastfeeding Healthy, Sick and Preterm Babies: Review
Source: Healthcare (Basel). 2024 Dec 2;12(23):2418. doi: 10.3390/healthcare12232418 (PMC11641371; doi:10.3390/healthcare12232418)
Supplement: Supplementary file 1 [file healthcare-12-02418-s001.zip › healthcare-3280835-supplementary.pdf]

Table S1. Overall characteristics of included studies.

Study	Study Design	Aims	Participants	Setting/Intervention
ACOG (2021) [88]	ACOG Committee opinion summary (review)	to compile existing barriers and supporting initiatives in the USA	position paper with recommendations	
Bennett; Grassley (2017) [84]	project description	to develop and implement a sustainable organizational process that would expedite support by providing WIC peer counsellors access to mothers of late preterm infants before hospital discharge		
Cantu et al. (2018) [73]	anonymous web-based survey	to assess and compare the most common barriers to successful breastfeeding perceived by female physicians in various stages of training and practice	223 female faculty and trainees in *Arkansas, USA*	quantitative questionnaires
Carpey et al. (2021) [27]	systematic review	identifying factors associated with successful breastfeeding in preterms and interventions to improve breastfeeding		n =11 studies
Crippa et al. (2019) [45]	prospective observational study	investigated factors and variables that could interfere with breastfeeding initiation and duration in preterm mothers	149 mothers and 189 neonates, including 40 pairs of twins in *Milan, Italy*	questionnaires on breastfeeding variables and habits to mothers of late preterm infants
Demirci et al. (2013) [44]	cohort study	evaluation of the prevalence of breastfeeding initiation and factors associated with breastfeeding non-initiation within a Pennsylvania population-based cohort of late preterm mother-infant dyads	late preterm mothers (n=62,451) and their infants (n=68,886), moderately preterm (n=17,325) and term (n=870,034) infants in *Pennsylvania, USA*	Comparison of breastfeeding initiation rates in 2003-2009
Desmond; Meaney (2016) [70]	qualitative study	to explore women's experiences of breastfeeding after their return to work	16 women *from Ireland*, contacted initially through a breastfeeding support website	Interviews and thematic analysis
Dunn et al. (2015a) [29]	qualitative study	to engage field-based professionals through a focus group process to collect perceptions on factors that determine a woman's decision to breastfeed	n = 43 health professionals in southern *New Hampshire, USA*	focus group interviews, thematic content analysis
Dunn et al. (2015b) [68]	cross-sectional design	assessment of barriers and positive contributors to breastfeeding initiation and duration	n = 283 women, infants, and children in southern *New Hampshire, USA*	Data from Special Supplemental Nutrition Program for Women, Infants, and Children (WIC) participants using the social ecological model
Ericson et al. (2018) [57]	randomised controlled trial	effectiveness of proactive telephone support provided to breastfeeding mothers of preterms after discharge from NICU	493 mothers of preterms in *Sweden*	the intervention group received a daily proactive telephone call up to 14 days after discharge from the support team. The control group could initiate telephone contact themselves

Study	Study Design	Aims	Participants	Setting/Intervention
Ericson; Palmér (2018) [58]	qualitative study	to describe how mothers of preterm infants experience breastfeeding support during the first 12 months after birth	151 mothers of preterms in *Sweden*	data from questionnaires with open-ended questions and telephone interviews were used
Estalella et al. (2020) [60]	quasi-experimental study (intervention later than control)	Evaluation of an intervention supporting breastfeeding program in a maternity service	mothers of late preterms from *Spain* n = 161 in intervention group, n = 212 in control group	intervention was designed to promote parents' education and involvement, provide a multidisciplinary approach and decision-making, and avoid separation of the mother-infant dyad
Gerhardsson et al. (2018) [52]	prospective, comparative study	investigation of breastfeeding self-efficacy in mothers of late preterm infants	mothers at 40 weeks of postmenstrual age (n = 148) and at three months of corrected age (n = 114) *in Sweden*	questionnaire (Breastfeeding Self-Efficacy Scale-Short Form)
Gianni et al. (2016) [49]	cross-sectional questionnaire survey	identify of facilitators and barriers to breastfeeding during hospital stays according to the experiences of mothers of late preterm infants	92 mothers who had given birth to 121 preterms in *Milan, Italy*	questionnaire
Gianni et al. (2018) [50]	cross-sectional questionnaire survey	identify of facilitators of and barriers to breastfeeding during hospital stay according to the experiences of mothers that gave birth to premature infants requiring admission to NICU	64 mothers who had given birth to 81 preterms (gestational age ≤33 weeks) in *Milan, Italy*	questionnaire
Golestani et al. (2024) [78]	online survey	to understand the experience of breastfeeding surgery residents and find opportunities for increased support	246 female US general surgeons who had given birth during residency in *Texas, USA*	questionnaire
González-Pascual et al. (2017) [83]	ethnographic study	to explore aspects related to the low rate of breastfeeding of immigrant Chinese mothers in Spain	8 Chinese mothers and 11 health workers in *Spain*	field observations and semi-structured informal interviews were conducted in two hospitals and a primary care centre
Goyal et al. (2014) [43]	cohort study	authors evaluated how hospital practices affect breastfeeding by gestational age	1,860 mothers in *USA*	questionnaire including questions about breastfeeding plans, hospital practices, and feeding patterns
Haas et al. (2020) [80]	review	presents an overview of relevant lactation physiology and evidence for specific strategies	-	review about lactation equipment and breast-pumping strategies
Hake-Brooks; Anderson (2008) [53]	randomized, controlled trial	determine the effects of kangaroo care on breastfeeding status	66 mothers and their preterm infants (32-36 completed weeks gestation) in *Utah, USA*	kangaroo care vs. standard nursery care
Hendrickson et al. (2022) [77]	cross-sectional survey	to determine the 6-month breastfeeding rates of pediatric emergency physicians, gain insight into their experiences expressing breast milk while	193 pediatric emergency physicians with 91 who had breastfed and 102 who	Survey with members of the American Academy of Pediatrics Section on Emergency Medicine via

Study	Study Design	Aims	Participants	Setting/Intervention
		working in pediatric emergency departments, and determine factors that support or discourage successful breastfeeding	had not in *Minnesota, USA*	its quarterly membership survey program.
Hookway et al. (2021) [51]	systematic review	challenges of breast/chestfeeding the medically complex child and to establish the gaps in healthcare provision that act as barriers to optimal infant and young child feeding	599 sick children from 7 countries	n = 11 studies included for review
Hookway; Brown (2023) [63]	mixed methods study	to explore the attitudes, awareness and perceived barriers to effective breastfeeding support provision, as well as healthcare professional perception of barriers for families	409 healthcare professionals (pediatric setting) in *Wales, GB*	Quantitative and qualitative data
Iliadou et al. (2018) [34]	quasi-experimental study with control group	evaluation of the effectiveness of a structured in-hospital midwife-led antenatal breastfeeding educational programme	203 nulliparous pregnant women in *Athens, Greece*	four-hour midwife-led antenatal breastfeeding educational programme on breastfeeding knowledge and self-efficacy, attitudes towards breastfeeding and perceived-barriers of breastfeeding
Jang; Hong (2020) [59]	quasi-experimental study	to investigate the effects of a breastfeeding support program on the prevalence of exclusive breastfeeding and growth in late-preterm infants	40 late preterm infants in *Korea*	web-based breastfeeding education program + practical support through home visits for mothers over 4 weeks in experimental group
Jónsdóttir et al. (2020) [28]	cohort study	breastfeeding progression, maternal feeding difficulties and associated factors for exclusive breastfeeding for late preterm and term infants	n = 60 late preterms from NICU, n = 60 late preterms from Maternity Unit and n = 369 term infants *in Iceland*	women completed questionnaires at several survey times
Kair; Colaizy (2016a) [39]	retrospective cohort study	identify barriers to breastfeeding continuation among overweight and obese mothers	19,145 mothers (3,717 (19%) were obese and 4,367 (23%) were overweight) in *Maine/ USA*	data from a nationally-representative survey
Kair; Colaizy (2016b) [47]	retrospective cohort study	to determine barriers to and facilitators of breastfeeding continuation among late preterm infants	2,530 mothers of late preterm infants	data from a nationally-representative survey
Keely et al. (2015) [38]	qualitative analysis comprising semi-structured face-to-face interviews	influencing factors of breast-feeding practices in obese women	28 obese women at 6-10 weeks following birth in *Scotland*	participants recruited from one large maternity unit
Kozhimannil et al. (2014) [37]	retrospective analysis	relationship between complex pregnancy (hypertension,	n = 2,400 who gave birth in 2011-2012 in a *US hospital*	data from a nationally-representative survey

Study	Study Design	Aims	Participants	Setting/Intervention
		diabetes, or obesity) and early infant feeding		
Kuhnly (2015) [62]	case study	how can breastfeeding be promoted for preterm twins?	1 mother of late preterm twins	description of the children's care plan questionnaires
MacVane et al. (2017) [72]	Web-based survey	to describe and quantify the parental leave experiences of a nationally representative sample of emergency physicians	464 male and female Emergency Medicine physicians from *Maine, Massachusetts, Missouri, Utah, Virginia, USA*	
Majee et al. (2016) [69]	qualitative research design	to examine workplace barriers and facilitators to breastfeeding in a small rural American community	14 mothers and 17 senior business managers from rural *Missouri, USA*	semi-structured interviews with major employers and low-income working breastfeeding mothers, and a focus-group with another group of employed and unemployed breastfeeding low-income mothers
Mallan et al. (2018) [40]	cross-sectional secondary analysis	comparison of self-reported breastfeeding problems in non-overweight and overweight women	n = 477 postpartum women in *Australia*	data collected as part of a hospital-based longitudinal study
Mattsson et al. (2015) [46]	comparative study (quantitative)	influence of supplementary artificial milk feeds on breast feeding and certain clinical parameters among healthy late preterm infants	parents of 77 late preterm infants in *Sweden*	data were collected via parental diaries
McFadden et al. (2017) [32]	Cochrane systematic review/ meta-analysis	effectiveness of breastfeeding support in mothers with healthy term babies	83,246 mother-infant pairs from 29 countries	review includes 100 trials of which 73 studies con-tribute data
Melnitchouk et al. (2018) [75]	online survey	to evaluate the barriers to breastfeeding for physicians who are mothers	1,606 female US physicians in *Massachusetts, USA*	quantitative questionnaire, large representative nationwide cohort
Michaud-Létourneau et al. (2022) [64]	qualitative study	barriers and potential solutions to improve the undergraduate training programs for various health professionals	n = 30 faculty and program directors from medicine, midwifery, nursing, nutrition and pharmacy (I.), n = 48 participants from the same disciplines, plus dentistry and chiropractic (II.) in *Québec, Canada*	focus group interviews (I.) und group discussion (II.)
Mitha et al. (2019) [55]	cohort study	analyse hospital unit characteristics and breast milk feeding policies associated with breast milk feeding at discharge	883 infants born at 32 to 34 weeks' gestation in *France*	data from French national cohort of preterm births (EPIPAGE-2)
Mörelius et al. (2015) [54]	randomized, controlled trial	effects of almost continuous skin-to-skin contact on salivary cortisol, parental stress, parental depression, and breastfeeding	47 families from two different neonatal care units in *Sweden*	skin-to-skin contact vs. standard care; effects were evaluated via questionnaires
Nanthakomon et al. (2023) [81]	cross-sectional study	to evaluate the incidence and factors related to exclusive	110 mothers from a nursing department in *Thailand*	questionnaire

Study	Study Design	Aims	Participants	Setting/Intervention
		breast feeding in the hospital personnel		
Niela-Vilén et al. (2016) [56]	randomized controlled trial	examination whether an internet-based peer-support intervention has an effect on the duration of breastfeeding or breast milk expression or maternal breastfeeding attitude compared with routine care	124 mothers of preterm (<35 weeks) infants in *Finland*	intervention was a closed peer-support group in social media. Data were collected by structured questionnaires
Nourse (2024) [79]	Cross-sectional online survey	to explore physician associate/assistant mothers' breastfeeding intention, duration, as well as workplace barriers for breastfeeding	545 physician associate/assistant mothers in *Washington, USA*	questionnaire
Patil et al. (2020) [22]	mixed-methods systematic review	identify barriers to exclusive breastfeeding among mothers	Not reported	44 studies were included for analysis
Ramírez-Durán et al. (2024) [65]	mixed-methods quasi-experimental design	to evaluate changes in students' breastfeeding knowledge	n = 40 nursing students in *Avila, Spain*	educational program with focus groups, a clinical simulation and a visit to the local breastfeeding association for students in intervention group
Rayfield et al. (2015) [42]	secondary analysis	association between breastfeeding support and breastfeeding among late preterm (gestation 34-36 weeks) and term (gestation ≥37 weeks) infants	14,525 term and 579 late preterm infants in *UK*	Questionnaire with breastfeeding support questions
Rykiel et al. (2023) [85]	quality improvement project	to investigate current breastfeeding practices and barriers to feeding for mothers	28 mothers in *Sarasota County, Florida, USA*	questionnaires
Sattari et al. (2013) [71]	quantitative interview study	to further explore physician mothers' personal infant feeding decisions and behaviour as well as their clinical breastfeeding advocacy	80 physician mothers with 152 children from 11 specialties in *Florida, USA*	questionnaires
Sayres; Visentin (2018) [87]	review	to review the recent literature on barriers to breastfeeding as well as strategies for pediatricians to use to help women overcome them	overall thematic review without structured research	
Schanler et al. (1999) [67]	feeding study with intervention- and control group	to describe the growth, nutritional status, feeding tolerance, and health of participating premature infants who were fed fortified human milk in comparison with those who were fed exclusively preterm formula	108 preterm infants in *Houston, Texas, USA*	the type of milk was determined by parental choice
Sriraman; Kellams (2016) [86]	review	To review the evidence related to barriers (prenatal, medical, societal, hospital, and sociocultural) that many mothers face, and explore the known barriers and the impact	overall thematic review without structured research	

Study	Study Design	Aims	Participants	Setting/Intervention
		they have on a woman's ability to breastfeed her infant		
Taylor (2023) [82]	systematic review	this review identifies barriers and enablers to accessing appropriate workplace breastfeeding facilities and the relevance of these factors to the British Army	16 studies	search strategy combined UK workplaces and English-speaking military breastfeeding studies in high-income countries, existing policy and guidance
Whiteside et al. (2020) [74]	mini-review	identify barriers to breastfeeding for emergency medicine physicians in the emergency department	7 studies	-
Wynn et al. (2021) [76]	secondary data analysis	to identify current breast-feeding policies available at orthopedic residency programs via a thorough review of individual programs websites	178 accredited orthopedic surgery residencies in, *USA*	residency program websites were analysed
Yang et al. (2019) [48]	qualitative descriptive study	develop an understanding of mothers' experiences breastfeeding a hospitalized preterm infant and the support needed to establish a milk supply during the period separation from their infants	11 Chinese mothers with preterm babies in *Beijing/ China*	qualitative interviews
Zhang et al. (2018) [33]	cross-sectional study	to investigate the related factors of exclusive breastfeeding based on the theory of planned behaviour	272 mothers in *Shanghai/ China*	data on mothers' breastfeeding knowledge, attitude, subjective norm and practice control were collected at 4 months postnatal
Zhang et al. (2024) [61]	quasi-experimental study	development of an online-based breastfeeding education and support program and exploration of its impacts on promoting mothers of preterm infants' breastfeeding knowledge, attitudes, self-efficacy, and rates	50 mothers of preterms in *Wuhan, China*	WeChat program for online instruction and support over 3 months (course, consultation, peer support and follow-up)

Abbreviations: ACOG = American College of Obstetricians and Gynecologists

Table S2. Main outcomes of included studies.

Study	Main Outcomes
ACOG (2021) [88]	A multidisciplinary approach that involves community, family, parents, and health care professionals will strengthen the support for parents and help them achieve their breastfeeding goals
Bennett; Grassley (2017) [84]	key strategies included creating a workable process; addressing barriers and stakeholder concerns; planning a pilot program to test the process; and inviting the WIC breastfeeding peer counsellors to tour the hospital, meet nursing staff and practice scripting their initial encounter with mothers
Cantu et al. (2018) [73]	97% percent of women with breastfeeding experience reported at least one perceived barrier to successful breastfeeding. Trainees identified more barriers compared with faculty physicians (median count 5 vs. 3, $p = 0.014$)

Study	Main Outcomes
Carpey et al. (2021) [27]	breastfeeding rates were lower in preterm mothers with lower socioeconomic status and single-parent households
Crippa et al. (2019) [45]	mothers with higher educational levels and previous positive breastfeeding experience had a longer breastfeeding duration. The negative factors for breastfeeding were the following: Advanced maternal age, Italian ethnicity, the feeling of reduced milk supply and having twins
Demirci et al. (2013) [44]	mothers of 35- and 36-week infants were slightly, but significantly ($p < 0.01$), more likely to initiate breastfeeding than mothers of 34-week infants. The probability of breastfeeding was dependent on the mother's socio-demographic status
Desmond; Meaney (2016) [70]	women noted that cultural attitudes in Ireland coupled with inadequate or inconsistent advice from health professionals posed the biggest challenge they had to overcome in order to achieve to 6 months exclusive breastfeeding
Dunn et al. (2015a) [29]	commonly reported themes were identified that negatively influence a mother's decision to breastfeed such as modesty/general discomfort to breastfeed in front of others, negative breastfeeding perceptions of family members, friends, boyfriends and co-workers, breastfeeding not being viewed as the societal "norm", and the availability of free formula samples
Dunn et al. (2015b) [68]	babies fed breastmilk are less likely to become ill (86% versus 74%; $p = 0.04$), and breastfeeding helps mothers bond with their babies more quickly than formula feeding (88% versus 72%; $p < 0.01$). Breastfeeding duration was significantly related to employment status; among women who breastfed for 6 months or longer, 15% were employed full-time, 30% worked part-time, and 55% indicated "other" such as unemployed or stay-at-home mother ($p = 0.01$).
Ericson et al. (2018) [57]	There were no differences between the groups for exclusive breastfeeding in both groups
Ericson; Palmér (2018) [58]	genuine support strengthens: the mothers described how they were strengthened by being listened to and met with respect, understanding, and knowledge. The support was individually adapted and included both practical and emotional support.<br>inadequate support: health professionals were controlling and intrusive which sometimes diminished the mothers
Estalella et al. (2020) [60]	infants in the control group were 50.7% exclusive breastfeeding, 37.8% breastfeeding, and, 11.5% formula feeding at discharge, whereas in the intervention group, frequencies were 68.4%, 25.9%, and 5.7%, respectively ($p=0.002$). Mothers in the intervention group were 2.66 times more likely to use the breast-pump after almost all or all feeds and 2.09 times more likely to exclusively breastfeed at discharge
Gerhardsson et al. (2018) [52]	the scores were higher in the 87% of mothers that exclusively breastfed when their babies reached 40 weeks (57.1 out of 70) than those who did not (41.4, $p = < 0.001$), indicating better self-efficacy
Gianni et al. (2016) [49]	at discharge, any human milk was fed to 94 % of infants. In the multivariate analysis, having expressed breast milk was independently associated with an increased risk of being fed with either any human milk or formula only (OR = 2.73, 95% CI 1.05-7.1, $p = 0.039$), whereas being encouraged to practice kangaroo mother care tended to have a protective effect (OR = 0.46, 95% CI 0.2-1.06, $p = 0.07$)
Gianni et al. (2018) [50]	at discharge, any breastfeeding was recorded in 66% of infants. Thirty percent of the mothers reported that they had experienced some obstacles to breastfeeding. Specifically, infants born to mothers who experienced difficulties in pumping breast milk (OR = 4.6; 95% CI 1.5-13.9) or in providing an adequate amount of milk to the infant (OR = 3.57; 95% CI 1.1-11.5) were at higher risk of being fed with formula at discharge
Golestani et al. (2024) [78]	67% of the 246 survey respondents stated that they did not have adequate time for pumping and 56% rarely had access to a lactation room. 69% of mothers reported a reduction in milk supply and 64% stated that the time constraints of residency shortened the total duration they breastfed. 59% of women did not feel comfortable asking to pump
González-Pascual et al. (2017) [83]	The rapid return to work and the popular phenomenon of sending babies back to China for grandparents to raise constitute barriers for breastfeeding. Although breastfeeding is common practice in China, in Madrid the prioritization of productivity over reproduction and the existence of the so-called "satellite babies" and transnational maternity make the establishment and maintenance of breastfeeding difficult
Goyal et al. (2014) [43]	overall breastfeeding rates among late preterm, early-term, and term infants were 87, 88, and 92 % ($p = 0.21$), late preterm versus term infants were less likely to exclusively breastfeed (39.8 vs. 62.3%,

Study	Main Outcomes
	p = 0.002). High hospital support was associated with increased exclusive breastfeeding (AOR 2.21 [95% CI 1.58-3.09])
Haas et al. (2020) [80]	four common types of breast pumps are mentioned
Hake-Brooks; Anderson (2008) [53]	kangaroo care dyads breastfed significantly longer (5.08 months vs 2.05 months), p = 0.003 and also more exclusively at each measurement, p = 0.047
Hendrickson et al. (2022) [77]	of those physicians who expressed milk at work, only 7.6% felt they "always" had sufficient time to pump; 32% felt they "always" had what they considered to be an appropriate location to pump
Hookway et al. (2021) [51]	themes included practical and psychological challenges of continuing to breastfeed in a hospital setting, complications of the condition making breastfeeding difficult, lack of specialist breastfeeding support from hospital staff and a lack of availability of specialist equipment to support complex breastfeeding
Hookway; Brown (2023) [63]	this study explored the barriers experienced by staff, as well as professionals' perception of parent barriers. These included lack of knowledge of how to help, a default formula culture, the pressure of maintaining strict fluid balance and breastfeeding being a low priority in the face of critical illness.
Iliadou et al. (2018) [34]	intervention group had a more positive attitude towards breastfeeding (73.5% vs. 66.1%, p<0.001), greater knowledge (14.6% vs. 13.1%, p<0.001), more breastfeeding self-efficacy (51.4% vs. 45.6%, p<0.001) and less perceived barriers (27.4% vs. 31.0%, p<0.001)
Jang; Hong (2020) [59]	exclusive breastfeeding was 5.18 times more common in the experimental group than in the control group (OR = 5.18, 95% CI 1.11~16.70).
Jónsdóttir et al. (2020) [29]	preterm infants are less likely to be exclusively breastfed after discharge from hospital than term infants; there were no significant differences between preterms in the NICU and those in the maternity unit
Kair; Colaizy 2016a [39]	overweight and obese women had higher odds of discontinuing breastfeeding because their babies were not satisfied by breast milk alone (overweight OR = 1.39, 95% CI: 1.16-1.68; obese: OR = 1.26, 95% CI: 1.03-1.54). Obese mothers had higher odds of discontinuing due to breastfeeding difficulties (OR = 1.29, 95% CI, 1.05-1.58) and infant jaundice (OR = 1.81, 95% CI, 1.26-2.60)
Kair; Colaizy (2016b) [47]	factors associated with increased odds of breastfeeding for ≥ 10 weeks were higher maternal education, mother being married, and normal maternal BMI
Keely et al. (2015) [38]	three major themes emerged as influencing factors: the impact of birth complications, a lack of privacy, and a low uptake of specialist breast-feeding support
Kozhimannil et al. (2014) [37]	women with complex pregnancy had 30% lower odds of intending to breastfeed (AOR = 0.71; 95% CI, 0.52-0.98)
Kuhnly (2015) [62]	strategies to support sustained breastfeeding in late preterm multiple birth infants include developing a family-centred feeding plan in collaboration with the medical team, assessing and supporting breastfeeding sessions, promoting lactogenesis with pumping or manual expression and activating a support system for families
MacVane et al. (2017) [72]	53% percent of women and 60% of men reported working in a setting with a formal maternity leave policy; however, 36% of women and 18% of men reported dissatisfaction with these policies. Most reported that other group members cover maternity-related shift vacancies; a minority reported that pregnant partners work extra shifts prior to leave
Majee et al. (2016) [69]	Lack of compliance with the new law, inadequate breastfeeding information for mothers, and lack of support from co-workers and supervisors emerged as the main barriers to successful workplace breastfeeding
Mallan et al. (2018) [40]	frequency of self-reported breastfeeding problems was similar across weight status groups. “Not enough milk” was the predominant reason for giving infant formula. Overweight women were more likely than non-overweight women to agree that infant formula was as good as breastmilk
Mattsson et al. (2015) [46]	infants who received regular supplementary artificial milk feeds experienced a longer delay before initiation of breast feeding, were breast fed less frequently and had longer hospital stays than infants exclusively breast fed from birth
McFadden et al. (2017) [32]	results of the analyses continue to confirm that all forms of extra support analysed together showed a decrease in cessation of 'any breastfeeding', which includes partial and exclusive breastfeeding

Study	Main Outcomes
Melnitchouk et al. (2018) [75]	nearly half (788 [49.1%]) of the participants reported that they would have breastfed for longer if their job had been more accommodating
Michaud-Létourneau et al. (2022) [64]	substantive improvements of undergraduate training programs for BF could be obtained by addressing challenges related to the insufficient, or lack of, (i) interactions among various HPs, (ii) opportunities for practical learning, (iii) specific standards to guide course content, (iv) real-life experience with counselling, and (v) understanding of the influence of attitudes on professional practice
Mitha et al. (2019) [55]	rates of breast milk feeding at discharge were higher with kangaroo care (adjusted OR 2.03; 95% CI: 1.01-4.10]), early involvement of parents in feeding support (1.94 [1.23-3.04]), unit training in a neurodevelopmental care programme (2.57 [1.18-5.60]), and in regions with a high level of BMF initiation in general population (1.85 [1.05-3.28])
Mörelius et al. (2015) [54]	100% in the intervention group were breastfeeding partly or exclusively at discharge, compared to 84.2% in the control group. More infants were breastfeeding partly or exclusively in the intervention group, compared to the control group at one and four months, but the differences did not reach significance
Nanthakomon et al. (2023) [81]	from multiple logistic regression models, perception of breastfeeding obstacles (aOR 1.55, 95% CI 1.27-1.90), breastfeeding behaviour (aOR 1.12, 95% CI 1.01-1.24), and support from health care system (aOR 0.84, 95% CI 0.72-0.97) remain the significant factors associated with successful exclusive breast feeding
Niela-Vilén et al. (2016) [56]	the duration of overall breastfeeding was on average 3·0 and 4·3 months in the experimental and control groups, respectively. The intervention had no effect on breastfeeding or expressing the breast milk or maternal breastfeeding attitude
Nourse (2024) [79]	Workplace support from colleagues and support staff was associated with longer exclusive breastfeeding duration and any breastfeeding duration. Adequate time and place for expression of breastmilk and support from supervising or collaborating physicians were associated with longer duration of breastfeeding
Patil et al. (2020) [22]	in total 32 barriers were grouped under individual, group and society level factors. Meta-analysis indicated that mothers who smoked had 2.49 times more odds of not exclusively breastfeeding than non-smoking mothers and mothers who had undergone caesarean section had 1.69 times more risk of cessation of exclusive breastfeeding than mothers who had a vaginal childbirth
Ramírez-Durán et al. (2024) [65]	the intervention group had a mean difference of 10 points in improvement (mean =10.53, SD = 2.20, min = 7, max = 14), whereas the control group had a mean of 6 points (mean = 6.80, SD = 3.03, min = 3, max = 13). The multiple linear regression explained the intervention's effect
Rayfield et al. (2015) [42]	mothers who reported receiving contact details for breastfeeding support groups had a higher likelihood of breastfeeding late preterm (adjusted ORs, aOR 3.14, 95% CI 1.40 to 7.04) and term infants (aOR 2.24, 95% CI 1.86 to 2.68) at 10 days and term infants at 6 weeks (aOR 1.83, 95% CI 1.51 to 2.22)
Rykiel et al. (2023) [85]	there is an evident need for improvement in the pipeline of expanding collaboration among breastfeeding community agencies in Sarasota County
Sattari et al. (2013) [71]	only 34% of the children were actually still breastfeeding at 12 months. In 43% of cases, physician mothers stated that breastfeeding cessation was due to demands of work
Sayres; Visentin (2018) [87]	the mode of delivery, mother's socioeconomic status, return to work, and prenatal breastfeeding education have been reported as factors that influence breastfeeding. Family-centred models for breastfeeding, peer support groups and technology have been studied as potential ways to help women meet their breastfeeding goals
Schanler et al. (1999) [67]	significantly better outcomes for preterm infants who were breastfed with breast milk in terms of time of discharge from hospital, incidence of NEC and late-onset sepsis
Sriraman; Kellams (2016) [86]	strategies will be discussed to address some of the most common barriers women face along with a list of resources that can be useful in this effort. Gaps in care and areas that need further research will be noted
Taylor (2023) [82]	barriers and enablers to access from 16 studies were described by three thematic areas: attitudes to breastfeeding, facility provisions and use of facilities
Whiteside et al. (2020) [74]	different dimensions of barriers to breastfeeding for female physicians as well as benefits associated with breastfeeding are mentioned

Study	Main Outcomes
Wynn et al. (2021) [76]	dedicated lactation facilities were mentioned for 3 (1.7%) programs. The average number of female attendings per program was two (range 0-19), and the average number of female residents per program was three (range 0-14)
Yang et al. (2019) [48]	mothers of preterm infants reported physically and mentally challenging breastfeeding experiences during the period they were separated from their babies
Zhang et al. (2018) [33]	results showed that higher scores of breastfeeding knowledge (OR = 1.09, 95% CI = 1.04-1.14), attitude (OR = 1.04, 95% CI = 1.00-1.09), subjective norm (OR = 1.22, 95% CI = 1.11-1.34) and practice control (OR = 1.11, 95% CI = 1.02-1.20) were associated with a higher rate of exclusive breastfeeding
Zhang et al. (2024) [61]	the breastfeeding knowledge significantly differed between the two groups but without differences between different time points and in the grouping*time interaction. Within the intervention group, significant improvements were observed from T0 to T2 and T1 to T2. No significant differences in breastfeeding attitudes, self-efficacy and breastfeeding rates were found between and within groups

Abbreviations: ACOG = American College of Obstetricians and Gynecologists; aOR = adjusted Odds Ratio; BF = breast feeding; CI = confidence interval; HP = health professional; NEC = necrotizing enterocolitis; OR = odds ratio; SD = standard deviation

Table S3. In-depth analysis of workplace barriers, US healthcare sector.

Study	Results	Cause of Cessation/ Options for Action	Remarks
Sattari et al. (2013) [71]	BF initiation: 97%<br>goal of ≥ 12 month reached for 34%	Work demands	Longer BF duration if engaged in BF promotion for their patients
MacVane et al. (2017) [72]	Formal maternity leave policy reported in their hospital: 53% (women), 60% (men)	Not satisfactory, as only very few (7%) compensate maternity leave with full salary/<br>formal policies, improved maternity leave, address BF issues	Less support from colleagues when it comes to take over shifts due to maternity leave
Cantu et al. (2018) [73]	57% never breastfed; those who did reported lack of time, no appropriate place to pump, unpredictable schedule, short maternity leave, long working hours		Trainees identified more barriers than faculty physicians
Melnitchouk et al. (2018) [75]	41.7% continued lactation to ≥ 12 mo pp, 28.0% reached their personal duration goal	Inadequate time, rigid schedules, insufficient space; used breast pumps in office (99.2%), lactation room (19.7%), call room (12.8%), their car (13.9%), empty patient rooms, bathrooms, locker rooms or closets (20.6%)/accommodating schedule, longer maternity leave, dedicated lactation space	
Wynn et al. (2021) [76]	Five (2.8%) had written policies, thirty-six (20%) had a link to a website with such policies, three (1.7%) mentioned dedicated lactation facilities		Orthopedic surgery has the lowest recruitment of women in all surgical fields and will need 117 years to reach gender parity
Hendrickson et al. (2022) [77]	For those who did breastfeed: return to full-time work after maternity leave: 87%, duration of BF for ≥ 6 mo: 90%, never handed over their pager: 71%, had less than 15 min to pump: 49%	Lack of access to lactation facilities, lack of adequate time; biggest stress: leaving critical patients	Working conditions vary substantially by physician specialty.<br>Very supportive environment in this population—<br>physicians set examples to support other mothers

Study	Results	Cause of Cessation/ Options for Action	Remarks
Golestani et al. (2023) [78]	No adequate time for pumping: 67%, rarely access to a lactation room: 56%, reduction in milk supply: 69%	Time constraints in residency, did not feel comfortable to ask for a pump/ protected pumping time, accessible lactation rooms with refrigerators for storage and comfortable seating, more understanding, increased parental leave	Accreditation Council for Graduate Medical Education (ACGME) allowed 6 weeks of parental leave in 2022
Nourse (2024) [79]	BF at birth: 96.3%, at 3 mo: 83.8%, at 6 mo: 78.1%, at 12 mo: 54.8% exclusive BF at birth: 77.9%, at 3 mo: 75%, at 6 mo: 42.6% PA mothers failed their BF goals	27.6% quit breastfeeding after returning to work because of workplace demands/ inadequate time and place for expression and support from supervising or collaborating physicians	Low response rate in this study

BF = breastfeeding; mo = months; PA = physical assistance; pp = post-partum.
